# Understanding the interfacial water structure in electrocatalysis

**DOI:** 10.1093/nsr/nwae241

**Published:** 2024-07-13

**Authors:** Guoshuai Shi, Tingyu Lu, Liming Zhang

**Affiliations:** Department of Chemistry, iChEM (Collaborative Innovation Center of Chemistry for Energy Materials), Shanghai Key Laboratory of Molecular Catalysis and Innovative Materials, Fudan University, China; Department of Chemistry, iChEM (Collaborative Innovation Center of Chemistry for Energy Materials), Shanghai Key Laboratory of Molecular Catalysis and Innovative Materials, Fudan University, China; Department of Chemistry, iChEM (Collaborative Innovation Center of Chemistry for Energy Materials), Shanghai Key Laboratory of Molecular Catalysis and Innovative Materials, Fudan University, China

## Abstract

The structure of interfacial water molecules plays a crucial role in modulating the electrochemical surface kinetics. This article provides an in-depth understanding of the water molecule structure inside the double layer and its main influencing factors at the molecular scale.

Aqueous electrocatalysis contributes greatly to the blueprint of carbon neutrality through directly converting renewable electricity to value-added chemical fuels. The electrocatalytic process mainly occurs in the electrode double layer (EDL), the microstructure of which determines the reaction kinetics at the solid/liquid interface in all electrochemical processes. Back in the last century, the Gouy-Chapman-Stern (GCS) model was established to describe the EDL, dividing it into three regions—the inner Helmholtz plane (IHP), outer Helmholtz plane (OHP) and diffuse layer [1]. While this model has been widely adopted and successfully interpreted experiments macroscopically [2], the detailed molecular structure and intermolecular connections were ignored in the EDL, resulting in failure to understand the catalytic mechanism and deviations from the model [3–5]. For instance, understanding the capacitive response of the Helmholtz layer requires one to clearly illustrate the orientation of chemisorbed water molecules on the electrode surface [3]. To this end, molecular-level understanding of the EDL and the role of solvent, e.g. water in aqueous electrolysis, in mediating ion-surface interactions is of particularly fundamental importance.

For aqueous electrolysis, the interfacial water molecules (IWMs) in the EDL are fully involved in surface chemical reactions and mass transfer processes. The accumulation features of IWMs, associating with relevant parameters, e.g. the concentration and orientation of IWMs, the connectivity of hydrogen bonds in EDL and the rigidity of IWMs, strongly affect the surface catalytic kinetics. Therefore, understanding the structure and dynamics of water molecules at the solid/liquid interface is certainly an important topic in electrochemical surface science. However, probing the IWMs is notoriously challenging due to the interference from bulk water and the complexity of surface environments at the solid/liquid interface. Thus far, several techniques have been implemented for such an investigation, including vibrational spectroscopy (e.g. infrared absorption [6]/Raman spectroscopy [7], sum-frequency generation spectroscopy [8] and THz spectroscopy [9]) and X-ray based spectroscopy [10]. In this perspective, we will summarize the recent breakthroughs in understanding the role of IWMs on electrocatalysis, and emphasize three key factors, electric field, local cations and surface adsorbed species, that potentially influence the structural dynamics of IWMs. At the end of the perspective, we will discuss the remaining challenges in this particular field, as well as an outlook for the future.

As the unique characteristic of electrochemical reactions, the strong electric field perpendicular to the electrode surface across the EDL is crucial in tuning the structure of IWMs due to the strong dipole moment of water molecules. By probing the secondary electrons at the oxygen K-edge, electron yield soft X-ray absorption spectroscopy (XAS) unraveled the much lower concentration of hydrogen bonds within the first two to three water layers than those in the bulk [10]. Within ∼1 nm of the electrode surface, half of the IWMs exhibited saturated hydrogen bonds, while the other half demonstrated broken hydrogen bonds. More importantly, the orientation of IWMs depended on the electric field across the EDL, which strongly disrupted the hydrogen bonding network in the interfacial layer. It is worthwhile to note that such soft X-ray–related measurements critically rely on a high vacuum, which poses a great challenge for the design and incorporation of *in-situ* electrochemical cells with the X-ray apparatus. In addition, X-rays can induce the radiolysis of liquid water, leading to water ionization with the formation of hydrated-electrons and cationic holes (H_2_O^+^) [11], which may also interfere with the study of IWMs.

The EDL contains solvated ions, which strongly perturbates the structure of IWMs and consequently the electrocatalytic reaction rate. A groundbreaking study on this topic was reported in 2021 [7]. By combining *in situ* shell-isolated nanoparticle-enhanced Raman spectroscopy with *ab initio* molecular dynamics simulations, two types of interfacial water structures were identified within 0.4 nm of the electrode surface, including hydrogen-bonded water and Na^+^-ion-hydrated water (Na·H_2_O). A structural ordering of IWMs (that is, an entropy decreasing process) was observed under negative potentials, which is conducive to the high-efficiency electron transfer, leading to higher hydrogen evolution reaction (HER) rates. Interestingly, through tracking the evolution of two types of water structures, a positive correlation between the reactivity and Na·H_2_O population was elucidated, indicating that the ordered Na·H_2_O structure boosted HER activity. Koper *et al.* also suggested that cations, such as Na^+^, play a key role in stabilizing the transition state of the rate-determining Volmer step in HER as proton donors by favorably interacting with the dissociating water molecules, forming water-ion clusters (*H–OH^δ−^-cat^+^) [12], which was mechanistically debated with that proposed by Chan and co-workers, wherein the IWMs and ions worked separately to improve the reactivity [13]. These observations highlight the pivotal role of the interfacial water structure, and prove the regulatory effect of cations. It is worthwhile to note that the type of solvated ions also determines the structure of hydration shells at the electrode surface. Using THz spectroscopy, Havenith *et al.* followed the stripping away of the cation/anion hydration shells in an NaCl electrolyte at Au surface [9]. Under low positive potentials, Cl^−^ maintains its full hydration layer with a growing number of hydrogen bonds, while Na^+^ directly loses part of its hydration shell at a low negative potential. Their observations went beyond predictions from the continuous GCS model, which describes the accumulation of ions in terms of the electrostatic interactions without accounting for the specific ion–water and water–water interactions at the interface.

Beyond the electric field and cations, surface adsorbed species, such as OH_ad_ (adsorbed OH group) [6], organic additives [14,15] and adsorbed reaction intermediates [8], could also influence the hydrogen-bond networks of IWMs within EDL, and tune the reactivity of water dissociation. A recent study employing surface-enhanced infrared absorption spectroscopy unveiled a hydrogen bond gap between OHP and the diffuse layer in an alkaline media on Pt, which impeded the transfer of protons and was responsible for the observed inferior HER activity in alkaline [6]. They further highlighted an unexpected contribution of OH_ad_ in boosting the alkaline HER kinetics through bolstering the connectivity of hydrogen-bond networks within the EDL, rather than solely affecting the energetics of surface reaction steps. Notably, another study relevant to the alkaline HER on Pt(100) solely using simulation, proposed that under high pHs, the negatively charged Pt tends to repel water adsorption, which in turn increases the apparent hydrogen binding energy, and therefore decreases the HER performance [16]. A similar pH-dependent activity has also been observed in the oxygen reduction reaction (ORR) [17], arising from the distinct IWM configurations in the EDL, which impacts the formation of hydrogen bonds between the oxygenated intermediates and IWMs, and eventually controls the kinetics of the proton-coupled electron transfer steps. Beyond OH_ad_, the adsorption of electrochemical intermediates may also influence the interfacial water structure. For instance, sum-frequency vibrational spectroscopy was implemented to investigate the structure evolution of IWMs on a graphitic electrode [8]. The hydrogen-bond network of IWMs remained intact without Faradaic electron transfer, indicating in that case, the structural evolution of IWMs strongly related to the intermediate adsorption, rather than the electric field.

The schematic diagram in Fig. 1 provides a summary of the recent progresses in understanding the role of IWMs in electrocatalysis and the key factors that influence the evolution of interfacial water structure. Beyond these three factors, some other parameters, such as the composition and atomic arrangement of catalysts, ionic liquids and surface adsorbed anions, etc., may play significant roles under certain circumstances, in tuning the configuration of IWMs, and eventually adjusting the electrocatalytic activity.

**Figure 1.**
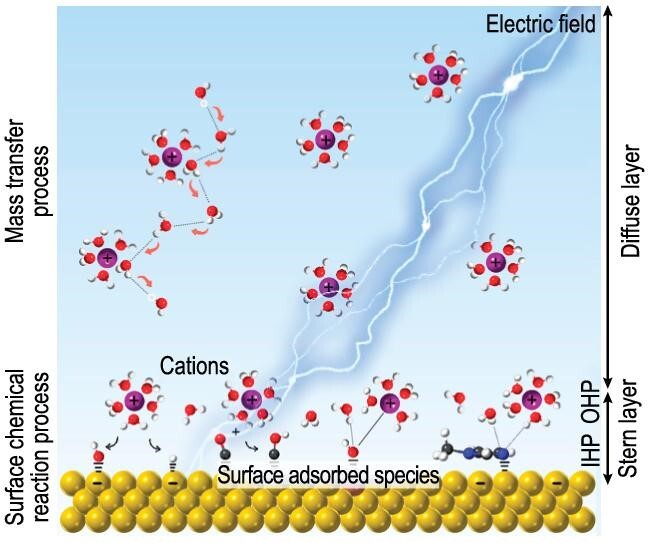
Interfacial water structure and its potential influencing factors in aqueous electrocatalysis.

Although considerable progress has been made, significant challenges remain, including the acquisition of high-quality signals of IWMs and the accurate attribution of experimental spectra. Further *operando* interface-sensitive technologies and home-customized electrochemical cells with specific architectures must be developed in order to eliminate the interference of bulk water. More advanced molecular dynamics calculations should also be developed in order to correlate the detailed structures of IWMs with the experimental spectra. Furthermore, it would be beneficial to investigate the IWMs in broader reaction systems, such as acidic water oxidation, carbon dioxide reduction and nitrogen reduction, which are more complex and challenging than HER. For instance, highly concentrated electrolytes, which disrupt the hydrogen bonding of IWMs and decrease the H_2_O activity, have been used to uncover the role of IWMs in controlling the branching between C_1_ and C_2+_ pathways in carbon dioxide reduction [18]. In a word, we believe the structure of IWMs offers a complementary descriptor beyond the adsorption energy, which is useful to guide the rational design of high-performance electrocatalysts and reaction microenvironments.
